# A Potent and Selective Peptide Blocker of the Kv1.3 Channel: Prediction from Free-Energy Simulations and Experimental Confirmation

**DOI:** 10.1371/journal.pone.0078712

**Published:** 2013-11-07

**Authors:** M. Harunur Rashid, Germano Heinzelmann, Redwan Huq, Rajeev B. Tajhya, Shih Chieh Chang, Sandeep Chhabra, Michael W. Pennington, Christine Beeton, Raymond S. Norton, Serdar Kuyucak

**Affiliations:** 1 School of Physics, University of Sydney, Sydney, New South Wales, Australia; 2 Department of Molecular Physiology and Biophysics, Baylor College of Medicine, Houston, Texas, United States of America; 3 Graduate Program in Molecular Physiology and Biophysics, Baylor College of Medicine, Houston, Texas, United States of America; 4 Medicinal Chemistry, Monash Institute of Pharmaceutical Sciences, Monash University, Parkville, Victoria, Australia; 5 Peptides International, Louisville, Kentucky, United States of America; University of Bologna & Italian Institute of Technology, Italy

## Abstract

The voltage-gated potassium channel Kv1.3 is a well-established target for treatment of autoimmune diseases. ShK peptide from a sea anemone is one of the most potent blockers of Kv1.3 but its application as a therapeutic agent for autoimmune diseases is limited by its lack of selectivity against other Kv channels, in particular Kv1.1. Accurate models of Kv1.x-ShK complexes suggest that specific charge mutations on ShK could considerably enhance its specificity for Kv1.3. Here we evaluate the K18A mutation on ShK, and calculate the change in binding free energy associated with this mutation using the path-independent free energy perturbation and thermodynamic integration methods, with a novel implementation that avoids convergence problems. To check the accuracy of the results, the binding free energy differences were also determined from path-dependent potential of mean force calculations. The two methods yield consistent results for the K18A mutation in ShK and predict a 2 kcal/mol gain in Kv1.3/Kv1.1 selectivity free energy relative to wild-type peptide. Functional assays confirm the predicted selectivity gain for ShK[K18A] and suggest that it will be a valuable lead in the development of therapeutics for autoimmune diseases.

## Introduction

High-affinity binding to the target protein is a key criterion in the search for drug leads, and several methods, from high-throughput screening to computational docking, have been used for this purpose. The selectivity of a drug lead for a given target is also important as high affinity for an unintended target could lead to unacceptable side effects. In principle, experimental or computational screening methods developed for lead searching can be used to address the selectivity problem, although the complexity of proteins compared to lead ligands makes such brute force methods less likely to be successful. Methods based on structure-based drug design provide a promising alternative for solving selectivity problems, as we illustrate in this study, which also identifies a potent analog of ShK peptide that is selective for the voltage-gated potassium channel Kv1.3.

Kv1.3 is a well-established target for the treatment of autoimmune diseases mediated by effector memory T_EM_ lymphocytes, such as multiple sclerosis and rheumatoid arthritis [1], [2]. ShK peptide, from the sea anemone *Stichodactyla helianthus* [3]–[5], is a potent blocker of the Kv1.3 channel with an IC_50_ of around 10 pM [6], and thus an excellent lead for development of an immunomodulatory drug [7]. However, ShK also blocks other Kv channels with high affinity, in particular Kv1.1 (IC_50_ = 16 pM) [6]. This lack of specificity for Kv1.3 is an impediment for the use of ShK as a therapeutic agent, and considerable effort has gone into developing ShK analogs with improved selectivity for Kv1.3 over Kv1.1 and other potassium channels [5], [7]. Several analogs with the desired selectivity have been developed, e.g. ShK-Dap22 [6], ShK-F6CA [8], ShK-186 [9], and ShK-192 [10], but all of them contain non-natural amino acids and/or adducts, and, in the case of ShK-186, the phosphorylated Tyr residue is susceptible to hydrolysis. For this reason, it is desirable to develop selective ShK analogs for Kv1.3 containing only natural amino acids.

An essential first step in searching for selective analogs using structure-based drug design methods is to construct accurate models of the relevant protein–ligand complexes. This has been achieved recently for Kv1.x–ShK complexes using docking and molecular dynamics (MD) simulations [11], [12]. The validity of the complex models was established by comparing the predicted binding modes with alanine scanning mutagenesis data [13], [14], and showing that the binding free energies of ShK obtained from the potential of mean force (PMF) calculations reproduce the experimental values within chemical accuracy [11]. Comparison of the binding modes in the Kv1.1–ShK and Kv1.3–ShK complexes reveals that the side chain of K18 in ShK is strongly coupled to a glutamate side chain in Kv1.1 via electrostatic interactions but it does not exhibit any interactions with the Kv1.3 residues [11]. Thus, mutation of this residue in ShK to alanine could lead to a substantial selectivity gain for Kv1.3 over Kv1.1.

Here we perform free energy simulations to calculate the change in the binding free energy of the Kv1.1–ShK and Kv1.3–ShK complexes associated with the K18A mutation. Two independent methods are used to check the veracity of the calculations. First we perform free energy perturbation (FEP) and thermodynamic integration (TI) calculations where ShK in the binding site is transformed to ShK[K18A] while the reverse transformation is carried out in the bulk for ShK[K18A], which gives directly the change in the binding free energy due to the mutation. We avoid convergence problems by using uncharged side chains as intermediate states. Convergence of the results was checked using the TI method, which also demonstrates the robustness of the calculations. Secondly, the PMFs for the dissociation of ShK[K18A] from Kv1.x are constructed from umbrella sampling MD simulations, from which the relative and absolute binding free energies of ShK[K18A] are determined. The change in the binding free energy is obtained by subtracting the relative binding free energy of the mutant from that of wild-type peptide [11]. The FEP and TI methods are computationally much cheaper but their reliability is not very well established for charge mutations. Thus, one of our aims was to demonstrate that the free energy change due to a charge mutation can be calculated accurately using the FEP and TI methods. The predictions of the free energy calculations were confirmed by measuring the binding constants of ShK[K18A] for Kv1.1 and Kv1.3 by patch-clamp electrophysiology.

## Materials and Methods

### Ethics statement

All procedures involving vertebrate animals were conducted after approval by the Institutional Animal Care and Use Committee at Baylor College of Medicine. Baylor College of Medicine follows the requirements of the Guide for the Care and Use of Laboratory Animals (National Research Council, 8th edition). Female Lewis rats were purchased from Taconic (Germantown, NY, USA) and housed in autoclaved setup with food and water *ad libitum* in an Association for Assessment and Accreditation of Laboratory Animal Care International (ALAAAC)-approved facility. Every effort was made to minimize animal discomfort and to keep the number of animals used to a minimum. Rats were euthanized by deep isoflurane anesthesia (slow breathing and lack of reactivity to toe pinching), followed by cardiac puncture. Death was ensured by decapitation.

### Peptide synthesis

The synthesis of the peptide has been described before [14]. Briefly, Fmoc-amino acid derivatives were obtained from Bachem A.G. (CH-4416 Bubendorf, Switzerland). Solid-phase assembly was initiated with an Fmoc-Cys(Trt)-2-chlorotrityl resin to minimize potential racemization of the C-terminal Cys residue. Automated stepwise assembly was carried out entirely on an ABI-431A peptide synthesizer (Applied Biosystems, Foster City, CA). ShK[K18A] was solubilized, oxidized, and purified by reverse phase-high pressure liquid chromatography using the method described previously [6], [13], and high pressure liquid chromatography-pure fractions were pooled and lyophilized. The structure and purity of the peptides were confirmed by reverse phase-high pressure liquid chromatography, amino acid analysis, and electrospray ionization-mass spectroscopy analysis. Samples were weighed and adjusted to account for peptide content before bioassay. The purity and mass were determined using LC and ESI-MS analyses (Figures S1 and S2 in File S1).

### NMR spectroscopy and data analysis

Samples were prepared by dissolving freeze-dried ShK[K18A] in 90% H_2_O/10% ^2^H_2_O, pH 4.8, to a concentration of 870 µM. One-dimensional ^1^H spectra and two dimensional homonuclear TOCSY spectra with a spin lock time of 80 ms were acquired at 20°C on a Bruker Avance 600 MHz spectrometer. A NOESY spectrum (mixing times 200 ms) was also acquired at 20°C, pH 4.8. All spectra were processed in TOPSPIN (version 3.0, Bruker Biospin) and analysed using CcpNmr-Analysis (version 2.1.5). ^1^H chemical shifts were referenced to the 1,4-dioxane signal at 3.75 ppm. Chemical shift assignments for backbone and side chain protons of ShK[K18A] were made by conventional analysis of TOCSY and NOESY spectra. A complete assignment of the proton NMR signals of ShK[K18A] was obtained.

The one-dimensional ^1^H NMR spectrum (Figures S3 and S4 in File S1) of ShK[K18A] showed sharp and well-dispersed resonances similar to that of wild-type ShK, indicating that the K18A mutation did not cause any significant perturbation of the native structure. To further compare the structures of wild-type ShK and the K18A mutant, chemical shift differences from random coil values for amide, H^α^ and H^β^ resonances were plotted (Figure S5 in File S1). This shows that the patterns of chemical shift deviations from random coil for ShK[K18A] closely resemble those for wild-type ShK, and confirms that the three-dimensional structures are basically the same. Small differences in chemical shifts between wild-type and mutant were observed only for resonances around the mutated residue K18 (Figure S5 in File S1).

### Cells

Mouse L929 fibroblasts stably expressing mKv1.1, mKv1.3, hKv1.5 channels [15] were gifts from Dr. K. George Chandy (University of California, Irvine). Human embryonic kidney 293 cells stably expressing hKv11.1 (hERG) were a gift from Dr. Craig January (University of Wisconsin, Madison). CCR7^−^ Ova-GFP T_EM_ cells (gift from Dr. Flügel, Munich, Germany) were derived from Lewis rats and react to ovalbumin [16]; they were maintained in culture through alternating rounds of antigen-dependent stimulation and of expansion in a cytokine-rich medium [17], [18]. As antigen-presenting cells for T_EM_ cell stimulation, we used irradiated (30 Gy) thymocytes harvested from humanely euthanized Lewis rats [17]. Histopaque-1077 gradients (Sigma-Aldrich) were used to isolate splenocytes from the same Lewis rats [9], [19].

### Electrophysiology

Electrophysiology experiments were conducted in the whole-cell configuration of the patch-clamp technique on a Port-a-Patch (Nanion Technologies, North Brunswick, NJ) connected to an EPC10-USB amplifier (HEKA Instruments, Bellmore, NY) [18], [20]. A holding potential of −80 mV was used for all recordings. Pipette resistances averaged 2.0 MΩ and series resistance compensation of 80% was employed when currents exceeded 2 nA. Kv1.3 currents were elicited by repeated 200-ms pulses from −80 mV to 40 mV, applied at intervals of 30 or 60 s. Kv1.3 currents were recorded in normal Ringer solution with a Ca^2+^-free pipette solution containing (in mM): 145 KF, 10 HEPES, 10 EGTA, 2 MgCl2, pH 7.2, 300 mOsm. IC_50_-values were determined by fitting the Hill equation to the reduction of area under the current curve measured at 40 mV. Kv1.1 and Kv1.5 currents were recorded with 200-ms depolarizing pulses to 40 mV applied every 10 sec. HERG (Kv11.1) currents were recorded with a 2-step pulse from −80 mV first to 20 mV for 2 sec and then to −50 mV for 2 sec and the reduction of both peak and tail current by the drug was determined.

### T Lymphocyte proliferation assays

The T_EM_ cells were used after 4 days of IL-2-dependent expansion. The splenic T cells were used immediately after collection from Lewis rats. Cells were seeded in flat-bottom 96-well plates in 200 µL of culture medium supplemented with 1% homologous rat serum. ShK or ShK[K18A] were added to the cells 30 min before the antigen or mitogen. T_EM_ cells were stimulated with 10 µg/ml ovalbumin in the presence of irradiated thymocytes as antigen-presenting cells and splenocytes were stimulated with 1 µg/mL concanavalin A. The cells were cultured for 3 days and were pulsed with [^3^H] thymidine (1 µCi/well) 16–18 h before harvest. The proliferative response of T cells was assessed in a β scintillation counter measuring the [^3^H] thymidine incorporated into their DNA [18], [20].

### Delayed type hypersensitivity

Lewis rats were immunized against ovalbumin [17] and a week later challenged in the pinna of one ear with ovalbumin or saline [17], [21]. They received a subcutaneous injection of 100 µg/kg ShK[K18A] at time of challenge and ear thickness was determined 24 h later as a measure of inflammation [9], [17], [18].

### Modeling of Kv1.x-ShK[K18A] complexes

The solution structure of ShK was obtained from the Protein Data Bank (PDB ID, 1ROO) [22]. Homology models of Kv1.1 and Kv1.3 were taken from previous work [11]. The structure of ShK[K18A] was generated from that of ShK using the VMD plugin, Mutator [23]. Two-dimensional homonuclear NMR spectroscopy was used to confirm that the solution structure of this analog was essentially identical with that of ShK [22], [24] (Figures S3, S4, S5 in File S1). The Kv1.x–ShK[K18A] complex models were generated via docking and refinement with MD following the same protocols used for Kv1.x–ShK complexes [11]. Briefly, the structure ShK[K18A] was equilibrated in bulk MD simulations for 5 ns, and ten frames were chosen for use in docking calculations. The initial Kv1.x–ShK[K18A] complexes were obtained using HADDOCK [25]. For both Kv1.1 and Kv1.3, the top ten poses with the highest scores were found to be very similar, and the one with the highest score was chosen for refinement with MD. Each complex structure was embedded in a lipid bilayer consisting of 125 POPC molecules and solvated with 10,525 water molecules and 0.1 M KCl. After the correct water and lipid densities were obtained with pressure coupling, the protein-toxin complex was gradually relaxed in 5-ns MD simulations. The equilibrated system was run for another 5 ns for analysis of the complex structure, where convergence of the channel protein and toxin peptide was monitored using the RMSD of the backbone atoms. MD simulations were performed using the NAMD program [26] with the CHARMM27 force field [27], which includes the CMAP correction [28]. An NpT ensemble was used with the temperature and pressure maintained at 300 K and 1 atm, respectively, via Langevin coupling with damping coefficients of 5 ps^−1^ and 10 ps^−1^. Periodic boundary conditions were employed together with particle-mesh Ewald algorithm to compute the long-range electrostatic interactions. Lennard-Jones (LJ) interactions were switched off within a distance of 10–12 Å, and the list of non-bonded interactions was truncated at 13.5 Å. A time step of 2 fs was used, and the trajectory data were written at 1 ps intervals.

### FEP and TI Calculations

**Figure 1:**
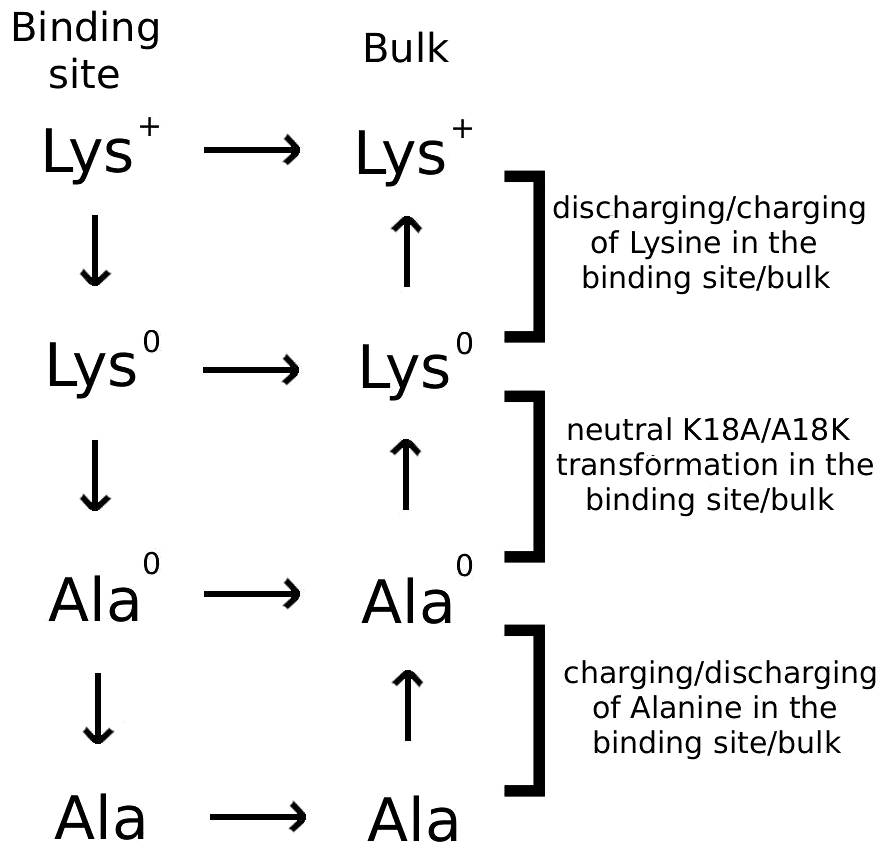
The thermodynamic cycle used in the free energy calculations. The superscript 0 denotes amino acids with no charges on the side chain atoms.

The free energy change due to a mutation is most efficiently calculated using the alchemical transformation methods such as FEP and TI [29], where the side chain of an amino acid is gradually transformed into another side chain and the free energy change is determined from the free energies of the end-points. This also applies to a protein-ligand complex, provided the binding mode is not altered by the mutation, which is the case for the K18A mutation in the Kv1.x–ShK complexes considered here. To avoid the problems posed by the change in the charge state, we implement the thermodynamic cycle in Figure 1. Accordingly, while the K18A mutation was applied to ShK in the binding site, the reverse transformation was applied to the toxin in the bulk simultaneously and in the same system, which ensures that the net charge in the system did not change during the calculations. To deal with convergence issues, we separated the Coulomb and LJ interactions and performed the K18A mutation in three stages as depicted in Figure 1. First, the K18 side chain on the bound ShK was discharged, while the reverse process was performed on ShK in the bulk with an uncharged K18 side chain. Secondly, the uncharged K18 side chain on the bound ShK was transformed to an uncharged A18 side chain, while the reverse was performed on the bulk ShK. Finally, the A18 side chain in the binding site was charged, while the corresponding side chain in the bulk was discharged (note that the Ala side chain is neutral overall but has small partial charges on C and H atoms). The change in the binding free energy due to the K18A mutation can, thus, be expressed as 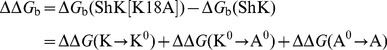
(1)


The first and last terms on the right hand side give the Coulomb contributions to the free energy change and the middle term gives the LJ contribution. Each term is calculated using the FEP and/or TI methods. In both methods, one introduces a hybrid Hamiltonian, 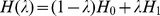, where *H*_0_ represents the Hamiltonian for the initial state and *H*_1_ for the final state. In the FEP method, the interval [0,1] is divided into *n* subintervals with [*λ_i_*, *i* = 1,…,*n* – 1], and for each subinterval the free energy difference is calculated from the ensemble average, 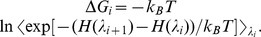 The free energy difference between the initial and final states is obtained from the sum, 
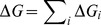
. Provided Δ*G_i_*≤2 kT for each window [27], the FEP sum should converge with reasonable sampling. For charge mutations, satisfaction of this condition would require over 130 windows if uniform subintervals were used. Using exponentially spaced subintervals instead halves the required number of windows to 66 (the *λ* values are provided in File S1). Each window was equilibrated for 80 ps followed by a 120-ps production run. These were found to be optimal times after several tests with different times. We also performed the backward calculation to check for hysteresis effects. The system was equilibrated for 5 ns after the forward transformation before starting the backward transformation. For the LJ part, we used 30 equally-spaced windows at the end points, i.e., for *λ* between 0–0.3 and 0.7–1, and 20 equally-spaced windows in between. Using 20-ps equilibration and 30-ps production run for each window was found to be adequate. In order to improve convergence and prevent instabilities, we used a soft-core LJ potential with a shift coefficient of 7.0 [30].

In the TI method, the ensemble average of the derivative, 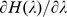, was obtained at several *λ* values, and the free energy difference was calculated from the integral, 
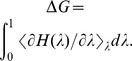
 The TI method was especially advantageous for charge interactions because Gaussian quadrature allows evaluation of the integral using a small number of windows, which can be sampled for longer times to check convergence of the results. A seven-point quadrature was found to be adequate in previous applications of the TI method [31], which was also adopted here (the *λ* values are provided in File S1). The initial TI windows were taken from the nearest FEP window. Each window was typically equilibrated for 1 ns and sampled for 1 ns. The TI method has not been used in the calculation of the LJ interactions because it offers no advantages over FEP (it requires as many windows due to the unsuitability of Gaussian quadrature and takes longer time), and good results have already been obtained using FEP.

### PMF Calculations

PMF provides the most reliable method for calculation of the absolute binding free energies of charged peptides, which can be directly compared to the experimental values. Unlike the FEP and TI methods, which give the free energy difference between two states, PMF provides a continuous free energy profile between the initial and final states. This makes PMF computationally more expensive than the FEP and TI methods. Nevertheless, it is very useful for testing of alternative methods. The PMFs for unbinding of ShK from Kv1.x are available from earlier work [11], and those for ShK[K18A] were constructed here using the umbrella sampling MD simulations employing the protocols developed in earlier work [11], [32]. Briefly, umbrella windows are generated at 0.5 Å intervals along the channel axis using steered MD simulations with *k* = 30 kcal/mol/Å^2^ and v = 5 Å/ns. After each pulling step, the toxin was equilibrated for 0.4 ns with the same restraining force to relax the effect of steering on the environment. The reaction coordinate was measured from the center of mass of the channel protein to the center of mass of the toxin along the *z* axis, which was aligned with the channel axis. A minimum of 5% overlap between the sampling of neighboring windows was required for an accurate construction of the PMF [32]. When the overlap was below this limit, e.g. due to a sharp rise in the PMF, an extra window was included between the two windows. The same umbrella force constant (30 kcal/mol/Å^2^) was used in all windows, which was found to be optimal for this size toxin [32]. The PMF was constructed from the reaction coordinates of ShK[K18A] collected during the umbrella sampling simulations via the weighted histogram analysis method [33]. Simulations were continued until the convergence of each PMF is assured. The binding constant was determined from the integral of the PMF, W(z), along the reaction coordinate, and the absolute binding energy from the log of the binding constant: 
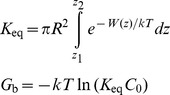
(2)


Here *z*_1_ is in the binding pocket and z_2_ is in the bulk where *W* vanishes. The factor *πR* ^2^ measures the average cross-sectional area of the binding pocket as explored by the center of mass (COM) of the toxin. The values of *R* were determined from the widths of the transverse fluctuations of the toxin COM. They were obtained from restraint-free MD simulations of the complexes as 0.71 and 0.70 Å, respectively, for Kv1.1–ShK[K18A] and Kv1.3–ShK[K18A]. C_0_ is the standard concentration of 1 M.

## Results and Discussion

### ShK[K18A] complexes with Kv1.1 and Kv1.3

**Figure 2:**
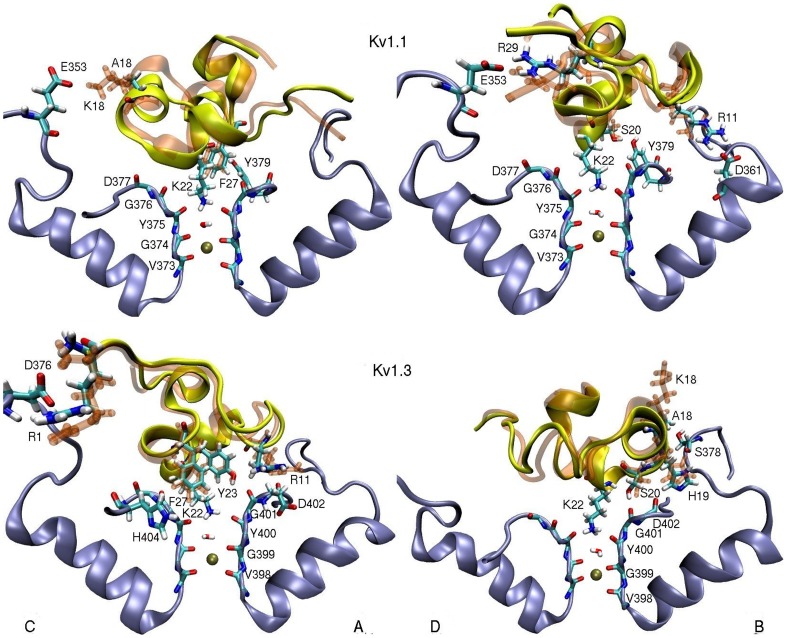
The K18A mutation does not change the binding modes of ShK to Kv1.1 and Kv1.3 channels. Snapshots of ShK[K18A] (yellow with colored side chains) and ShK (transparent orange) in complex with Kv1.1 and Kv1.3 are superposed to show that there is a good overlap between the toxin backbones, and all the important interactions identified in the ShK complexes are preserved in the ShK[K18A] complexes. To give the full picture, two views of the cross sections of the complex, depicting the monomers C and A (left panel) and D and B (right panel) are presented. Only the residues involved in binding are indicated explicitly.

Equilibrated structures of the Kv1.1–ShK[K18A] and Kv1.3–ShK[K18A] complexes were obtained as described in Methods. To visualize the effect of the K18A mutation on the binding modes, we superimpose the snapshots of the Kv1.x–ShK[K18A] complexes with those of ShK (Figure 2). In both complex structures, there is a very good overlap between the backbones of the mutant and wild-type ShK, indicating that the K18A mutation causes minimal perturbation of the binding mode. This observation is made more quantitative in Table 1, which compares the average interatomic distances between the strongly interacting residues in the mutant and wild-type complexes. All the contact pairs are seen to be preserved after the mutation in both complexes, and the changes in the pair distances are well within the fluctuations for all the strongly coupled pairs (distance < 4 Å).

**Table 1:** Comparison of the strongly interacting pair distances in the ShK–Kv1.x complexes with those in the ShK[K18A] –Kv1.x complexes.

ShK	Kv1.1	MD average	ShK[K18A]	Kv1.1	MD average
R11-N_2_	D361-O_2_(B)	5.5±0.5	R11-N_2_	D361-O_1_(B)	4.5±0.3
K18-N_1_	E353-O_2_(C)	2.7±0.2			
S20-O_H_	Y379-O_H_(B)	3.0±0.3	S20-O_H_	Y379-O_H_(B)	3.0±0.3
K22-N_1_	Y375-O(ABC)	2.7±0.2	K22-N_1_	Y375-O(BCD)	2.7±0.5
F27-C_ε2_	Y379-C_ε1_(A)	3.6±0.2	F27-C_ε2_	Y379C_ε1_(A)	4.0±0.4
R29-N_2_	E353-O_2_(D)	2.5±0.3	R29-N_2_	E353-O_2_(D)	2.8±0.4

ShK	Kv1.3	MD average	ShK[K18A]	Kv1.3	MD average
R1-N_1_	D376-O_1_(C)	4.5±0.4	R1-N_1_	D376-O_1_(C)	3.5±0.3
R11-N_2_	D402-O_1_(A)	3.5±0.3	R11-N_2_	D402-O_1_(A)	4.0±0.3
H19-N_H_	S378-O(B)	3.0±0.5	H19-N_H_	S378-O(B)	3.5±0.5
S20-O_H_	G401-O(B)	2.7±0.2	S20-O_H_	G401-O(B)	2.7±0.2
K22-N_1_	Y400-O(ABD)	2.7±0.2	K22-N_1_	Y400-O(ABD)	2.7±0.2
Y23-O_H_	G401-O(A)	3.5±0.3	Y23-O_H_	G401-O(A)	3.5±0.3
F27-C_ε1_	H404-C_γ_(C)	3.6±0.2	F27-C_ε1_	H404-C_γ_(C)	3.7±0.4

The average atom-atom distances obtained from 5 ns MD simulations are listed in column 3 for the wild-type complexes and in column 6 for the mutant complexes (in units of Å). Subscripts refer to the side chain atoms and the monomer identity in K^+^ channels is given in parentheses.

### Free energy changes from FEP and TI calculations

**Figure 3:**
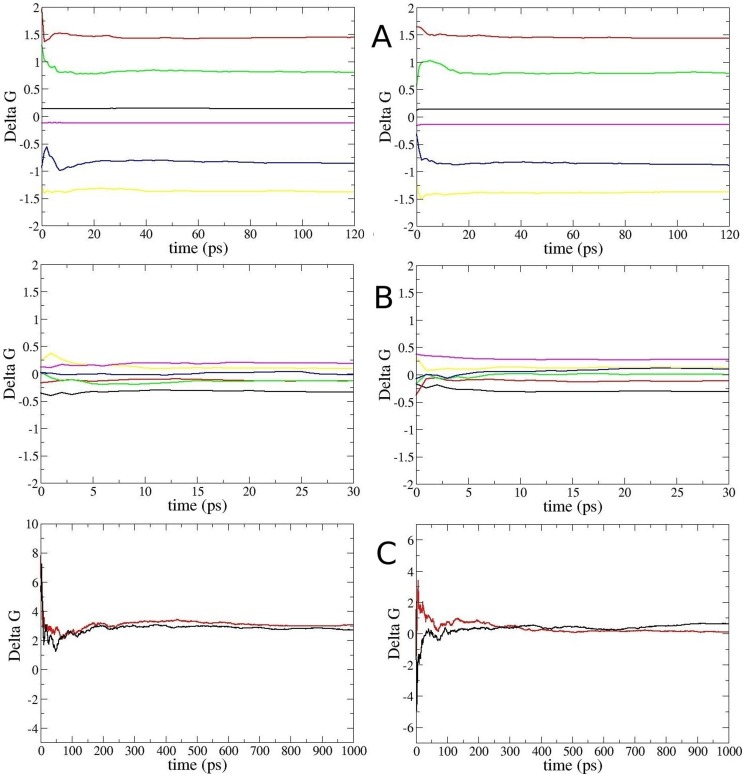
Convergence of the FEP and TI calculations. **(A)** Convergence of the FEP calculations for discharging/charging of the K18 in ShK in the binding site/bulk. Running averages of *ΔG_i_* for windows at λ = 0 (black), λ = 0.2 (red), λ = 0.4 (green), λ = 0.6 (blue), λ = 0.8 (yellow) and λ = 0.999 (magenta) are plotted as a function of the production time for Kv1.1 (left) and Kv1.3 (right). **(B)** Similar to A but showing the convergence of the FEP calculations for transformation of the uncharged Lys side chain to that of Ala. **(C)** Convergence of the TI calculations for discharging/ charging of K18 in ShK in the binding site/bulk. Running averages of the *ΔG* values obtained from the TI calculations are plotted as a function of the production time for Kv1.1 (left) and Kv1.3 (right). Both the forward (black) and the negative of the backward (red) results are shown to check for hysteresis effects, which remain well below 1 kcal/mol for both channels.

Provided the binding mode is preserved, the most straightforward way to calculate the binding free energy change due to a mutation on a ligand is to use the FEP and/or TI methods. To facilitate convergence, we adopted a staged approach, as described in Figure 1. We used both methods for the charging/discharging steps where convergence issues are critical for the long-range Coulomb interactions. Evidence for the convergence of the FEP and TI calculations for charging/discharging of Lys side chain is provided in Figure 3. Mutation of the uncharged Lys to Ala involves short-range LJ interactions, where convergence is not an issue provided the windows at the end points are chosen at small intervals. Because the TI method offers no advantages, we have performed three independent FEP calculations for the LJ step. Convergence of the FEP calculations for the LJ step is demonstrated in Figure 3B. Detailed results of the FEP and TI calculations showing the individual contributions from the three steps in both forward and backward directions for the Kv1.1 and Kv1.3 channels are presented in Table 2. We note that there is negligible hysteresis between the forward and backward calculations in all cases, and the FEP and TI calculations yield consistent results for the charging/discharging steps. As expected, the main contribution to the free energy difference comes from the charging/discharging of the Lys side chain, while the charging/discharging of the Ala side chain makes a negligible contribution.

**Table 2:** Differences in the binding free energy of ShK to Kv1.1 and Kv1.3 due to the K18A mutation, calculated with the FEP and TI methods.

Kv1.1/FEP	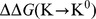	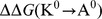	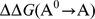	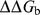
Forward	2.4±0.4	−0.3±0.1	−0.1±0.1	2.0±0.4
Backward	−2.5±0.5	0.3±0.1	0.1±0.1	−2.1±0.5
Average	2.5±0.5	−0.3±0.1	−0.1±0.1	2.1±0.5
**Kv1.3/FEP**				
Forward	0.9±0.3	−0.2±0.1	0.0±0.1	0.7±0.2
Backward	−0.4±0.3	0.2±0.1	0.0±0.1	−0.2±0.2
Average	0.7±0.3	−0.2±0.1	0.0±0.1	0.5±0.2
**Kv1.1/TI**				
Forward	2.7±0.5	−0.3±0.1	−0.1±0.1	2.3±0.5
Backward	−3.0±0.6	0.3±0.1	0.1±0.1	−2.6±0.6
Average	2.8±0.6	−0.3±0.1	−0.1±0.1	2.4±0.6
**Kv1.3/TI**				
Forward	0.6±0.5	−0.2±0.1	0.0±0.1	0.4±0.5
Backward	−0.1±0.5	0.2±0.1	−0.1±0.1	0.0±0.5
Average	0.4±0.5	−0.2±0.1	0.0±0.1	0.2±0.5

The three contributions to the binding free energy difference and their sum (eq. 1) are listed in columns 2–4 (in units of kcal/mol). In each case, the results of the forward and backward calculations are given separately, followed by their average. The TI calculations are performed for the Coulomb parts only; the LJ contribution is taken from the FEP calculations. The uncertainties are calculated from block data analysis of the data.

### Free energy changes from PMF calculations

**Figure 4:**
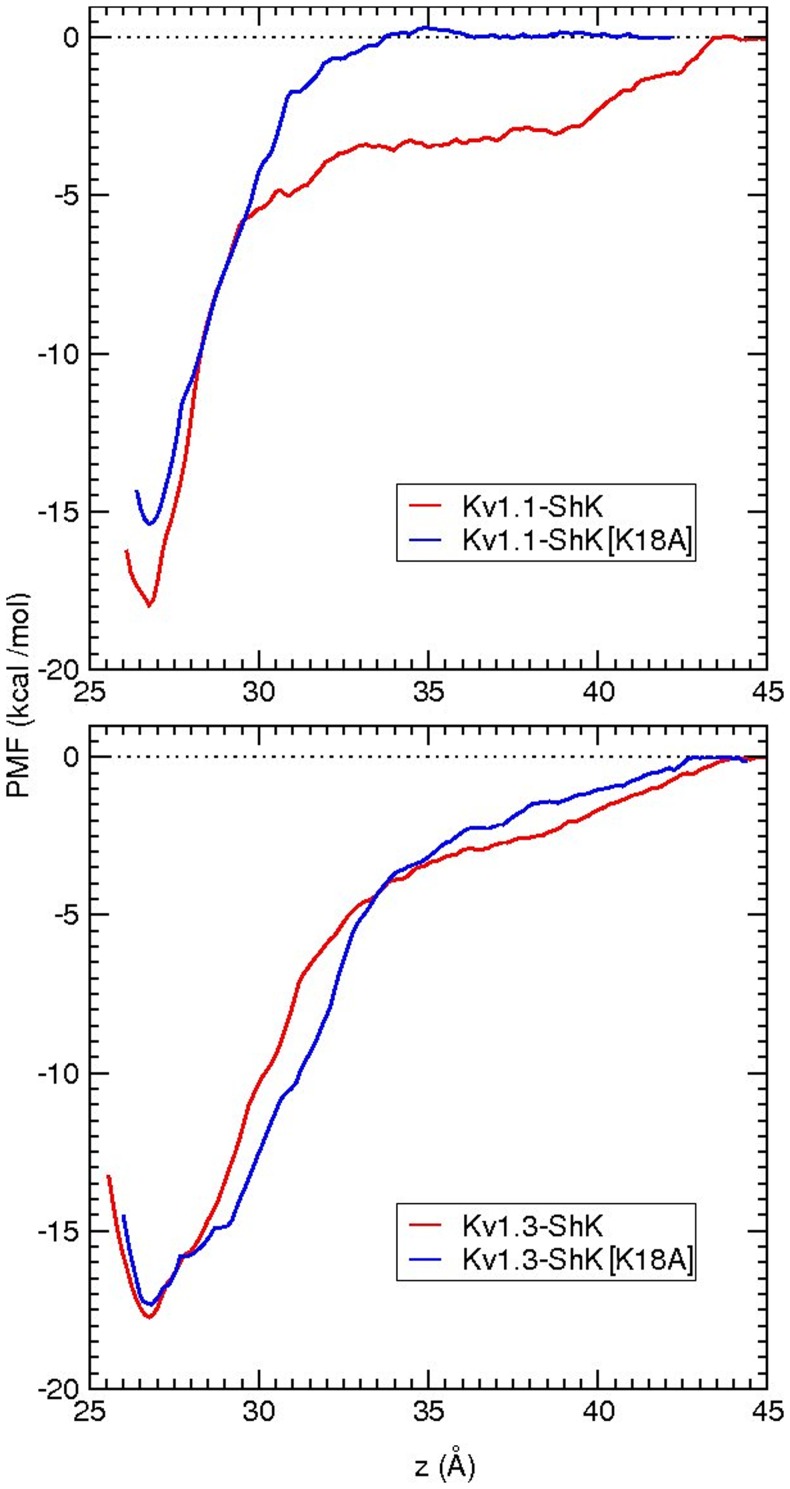
Comparison of the PMFs for the unbinding of ShK and ShK[K18A] from the Kv1.1 and Kv1.3 channels.

PMF calculations were then performed to check the accuracy of the FEP and TI calculations. The binding constants and absolute binding free energies obtained from PMFs also allow a direct comparison with the experimental values. The PMFs for the unbinding of ShK from Kv1.1 and Kv1.3 were constructed in our earlier work [11]. Here we repeat these PMF calculations for ShK[K18A] using the same protocols. Convergence studies of the PMFs from block data analysis (Figures S6 and S7 in File S1) show that the PMF in the Kv1.3–ShK[K18A] system converges relatively quickly, just as in the case of the Kv1.3–ShK system [11], and the final PMFs for ShK and ShK[K18A] are very similar (Figure 4). Convergence of the PMF in the Kv1.1–ShK[K18A] system is delayed somewhat (Figures S6 and S7 in File S1), which can be traced to the slow equilibration of the E353–R29 side chain interactions. As expected, abolishing a charge interaction in the Kv1.1–ShK[K18A] system leads to a shallower PMF compared with that for Kv1.1–ShK. An interesting feature of the ShK[K18A] PMF is the absence of the shoulder region observed in the ShK PMF, which is caused by the long-range Coulomb interactions, in particular by the E353–R29 pair [11]. To understand this feature, we compare the E353(O_2_)–R29(N_2_) pair distances in ShK and ShK[K18A] PMFs as a function of the channel-toxin distance, *z* (Figure S8 in File S1). The pair distance remains around 7–8 Å for *z*>35 Å in the ShK PMF but becomes double that in the ShK[K18A] PMF. Thus, there are no discernible charge interactions left after *z* = 35 Å in the ShK[K18A] PMF, which explains its earlier flattening compared to the ShK PMF. Similarly the bump in the Kv1.1–ShK PMF around *z* = 43 Å can be explained by the re-engagement of the E353–R29 pair due to rotation of the toxin and its subsequent dissociation (Figure S8 in File S1). Absolute binding free energies obtained from the integration of the PMFs (eq 2) are compared to the experimental values in Table 3. In all cases, the calculated binding free energies agree with the experimental values within the chemical accuracy of 1 kcal/mol, which provides strong support for the accuracy of the complex models.

**Table 3:** The relative and absolute binding free energies obtained from the PMF calculations for the Kv1.x–ShK and Kv1.x–ShK[K18A] complexes.

Complex	Δ*W*_well_	*G*_b_(PMF)	*G*_b_(exp)
Kv1.1–ShK	−18.0±0.7	−14.3±0.6	−14.4±0.1
Kv1.1–ShK[K18A]	−15.3±0.7	−11.7±0.7	−11.3±0.1
Kv1.3–ShK	−17.8±0.8	−14.2±0.7	−15.0±0.1
Kv1.3–ShK[K18A]	−17.4±0.7	−13.9±0.6	−14.2±0.1

The relative binding energy, Δ*W*_well_ is given by the well depth in the PMF and the absolute binding free energy, *G*_b_, is obtained from the integration of the PMF via eq. 2 (in units of kcal/mol). Experimental *G*_b_ is determined from the binding constants using eq. 2.

### Selectivity free energies

The results of the binding free energy differences obtained using various approaches are summarized in Table 4 together with the experimental values. The FEP and TI results are in good agreement with those of the PMF, which indicates that the former methods can be used reliably in calculation of the free energy differences associated with mutation of amino acids. The TI method gives slightly better results compared with FEP for charge interactions, presumably because of longer sampling of windows. The crucial question of the selectivity free energy gain for Kv1.3/Kv1.1 associated with the K18A mutation is addressed in the last column of Table 4. All methods predict a gain in selectivity free energy of about 2 kcal/mol, which is confirmed with the binding constant measurements of ShK[K18A] for Kv1.1 and Kv1.3.

**Table 4:** Comparison of the binding free energy differences for Kv1.1 and Kv1.3, and the selectivity free energy for Kv1.3/Kv1.1, obtained using the FEP, TI and PMF methods, with the experimental results.

	ΔΔ*G*_b_(Kv1.1)	ΔΔ*G*_b_(Kv1.3)	ΔΔ*G*_sel_
FEP	2.1±0.5	0.5±0.2	1.6±0.6
TI	2.4±0.6	0.2±0.5	2.2±0.8
PMF	2.6±1.0	0.3±1.0	2.3±1.4
Exp.	3.1±0.1	0.8±0.2	2.3±0.2

The FEP and TI results are taken from Table 2, and the PMF results are obtained from Table 3 using ΔΔ*G*_b_ = *G*_b_(ShK[K18A])−*G*_b_(ShK) (a similar result is obtained if Δ*W*_well_ is used instead of *G*_b_). The change in selectivity free energy due to the mutation in column 4 follows from ΔΔ*G*_sel_ **= **ΔΔ*G*_b_(Kv1.1)−ΔΔ*G*_b_(Kv1.3). All energies are in units of kcal/mol.

### Experimental validation of modeling results

**Figure 5:**
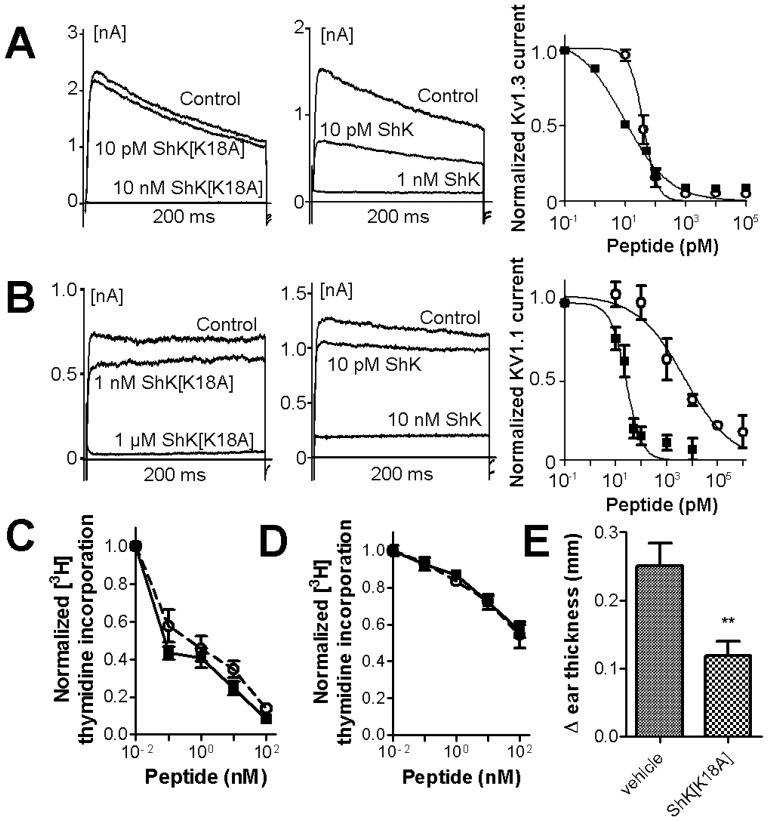
ShK[K18A] is selective for Kv1.3 over Kv1.1 channels and preferentially targets T_EM_ lymphocytes *in vitro* and *in vivo*. (A) Effects of ShK (▪) and ShK[K18A] (○) on Kv1.3 currents measured by whole-cell patch-clamp on L929 fibroblasts stably transfected with mKv1.3. The two left panels show whole-cell Kv1.3 currents before (control) and after perfusion of ShK[K18A] (left panel) or ShK (middle panel). The panel on the right shows the Kv1.3 currents remaining after steady-state block is reached with the different concentrations of ShK and its analog, fitted to a Hill equation (N = 5–6 cells per concentration).(B) Effects of ShK (▪) and ShK[K18A] (○) on Kv1.1 currents measured by whole-cell patch-clamp on L929 fibroblasts stably transfected with mKv1.1. The two left panels show whole-cell Kv1.1 currents before (control) and after perfusion of ShK[K18A] (left panel) or ShK (middle panel). The panel on the right shows the Kv1.1 currents remaining after steady-state block is reached with the different concentrations of ShK and its analog, fitted to a Hill equation (N = 6–7 cells per concentration).(C) Effects of ShK (▪) and ShK[K18A] (○ and dashed line) on the proliferation of rat T_EM_ cells measured *in vitro* by the incorporation of [^3^H] thymidine into the DNA of dividing cells (N = 3). (D) Effects of ShK (▪) and ShK[K18A] (○ and dashed line) on the proliferation of rat splenocytes (mainly naïve/T_CM_cells; N = 3).(E) Effects of the subcutaneous administration of 100 µg/kg ShK[K18A] on an active DTH reaction elicited against ovalbumin. Data show the difference in challenged and non-challenged ear thickness in vehicle-treated rats and ShK[K18A]-treated rats (N = 6/group).

In order to validate the modeling results by experimentation, we used the well-established technique of whole-cell patch-clamp. Using this technique, mouse fibroblasts stably expressing either Kv1.1 or Kv1.3 channels were patch-clamped to measure total potassium currents through these channels. The perfusion of various dilutions of ShK or ShK[K18A] induced Kv1.1 or Kv1.3 channel block, measured by a steady-state reduction in potassium currents through the Kv1 channels, allowing for the determination of the half-inhibitory concentration (IC_50_) for ShK and its analog. ShK[K18A] inhibited Kv1.3 channels with an IC_50_ of 39.6±3.8 pM, displaying a modest decrease in potency compared to ShK (IC_50_ on Kv1.3 = 9.3±2.0 pM) (Figure 5A) [20]. ShK also inhibited Kv1.1 channels with a high affinity (IC_50_ = 25.6±2.8 pM) (Figure 5B). In contrast, ShK[K18A] exhibited a 124-fold selectivity for Kv1.3 over Kv1.1 channels (IC_50_ on Kv1.1 = 4,900±200 pM).

A potential concern when developing potassium channel blockers is off-target toxic effects on cardiac channels. We therefore used the whole-cell patch-clamp assay to determine the effects of ShK[K18A] on the two cardiac potassium channels Kv1.5 and Kv11.1 (hERG). ShK[K18A] had no effects on either cardiac potassium channels when tested at concentrations of up to 100 nM (results not shown).

Since selective Kv1.3 channel blockers are known to preferentially inhibit the proliferation of CCR7^−^ T_EM_ lymphocyte with little to no effect on the proliferation of CCR7^+^ naïve and T_CM_ lymphocytes, we next tested the effects of ShK and ShK[K18A] on the proliferation of rat T lymphocytes. Both peptides displayed a marked preferential inhibition of the proliferation of T_EM_ lymphocytes (Figure 5C) with little effect on naïve/T_CM_ cells (Figure 5D). These results suggest that ShK[K18A] is selective for Kv1.3 over KCa3.1 channels as naïve/T_CM_ cells rely on KCa3.1 channels for their proliferation [1].

Finally, we used an active DTH reaction to ovalbumin to determine the *in vivo* ability of ShK[K18A] to inhibit a T_EM_ lymphocyte-mediated inflammatory reaction. ShK[K18A] reduced this active DTH reaction by *ca* 52% (Figure 5E), exhibiting similar activity to ShK-186 and ShK-192 in this model [10].

These results demonstrate that ShK[K18A] is bioavailable and can inhibit T_EM_ cells both *in vitro* and *in vivo*, making it a compound of considerable interest for immunomodulation and treatment of T_EM_-mediated diseases.

## Conclusions

Increasing the selectivity of a drug lead for a specified target is a design problem that in principle could be greatly facilitated using computational methods. Here we have described the case of ShK peptide, which binds with high affinity to the Kv1.3 channel and is a potential drug lead for the treatment of autoimmune diseases. We have analyzed the K18A mutation in ShK, which was suggested by accurate models of Kv1.x–ShK complexes, and shown via free energy calculations, to produce a 2 kcal/mol gain in selectivity free energy for Kv1.3/Kv1.1. Experimental confirmation of this result increases confidence in the ability of the free energy methods, in particular computationally cheaper FEP and TI methods, to predict the effects of mutations accurately. Moreover, the potency of ShK[K18A] against Kv1.3, its selectivity for this channel over Kv1.1, its preferential inhibition of T_EM_ cells over naïve and T_CM_ cells, its ability to significantly reduce a DTH reaction, and the fact that it is composed entirely of protein amino acids, make it an attractive candidate for further evaluation as a therapeutic for autoimmune diseases.

## Supporting Information

File S1 Figure S1, The LC profile of ShK[K18A]; Figure S2, ESI-MS analysis of ShK[K18A]; Figure S3, The 1D ^1^H NMR spectrum of wild-type ShK and ShK[K18A]; Figure S4, The amide and aromatic region of ^1^H NMR spectra of wild-type ShK and ShK[K18A]; Figure S5, Deviation from random coil chemical shifts of the H^N^, H^α^, and H^β^ resonances of wild-type ShK and ShK[K18A]; Figure S6, Convergence of the Kv1.x–ShK[K18A] PMFs from 2 ns block data analysis; Figure S7, Convergence of the Kv1.x–ShK[K18A] PMFs from 0.5 ns block data analysis; Figure S8, The E353(O_2_)–R29(N_2_) pair distances in ShK and ShK[K18A] PMFs.
(DOCX)
