# Supplementary material for: A Potent and Selective Peptide Blocker of the Kv1.3 Channel: Prediction from Free-Energy Simulations and Experimental Confirmation
Source: PLoS One. 2013 Nov 7;8(11):e78712. doi: 10.1371/journal.pone.0078712 (PMC3820677; doi:10.1371/journal.pone.0078712)
Supplement: File S1 — Figure S1, The LC profile of ShK[K18A]; Figure S2, ESI-MS analysis of ShK[K18A]; Figure S3, The 1D 1H NMR spectrum of wild-type ShK and ShK[K18A]; Figure S4, The amide and aromatic region of 1H NMR spectra of wild-type ShK and ShK[K18A]; Figure S5, Deviation from random coil chemical shifts of the HN, Hα, and Hβ resonances of wild-type ShK and ShK[K18A]; Figure S6, Convergence of the Kv1.x–ShK[K18A] PMFs from 2 ns block data analysis; Figure S7, Convergence of the Kv1.x–ShK[K18A] PMFs from 0.5 ns block data analysis; Figure S8, The E353(O2)–R29(N2) pair distances in ShK and ShK[K18A] PMFs. (DOCX) [file pone.0078712.s001.docx]

**Supporting Information File S1**

**A Potent and Selective Peptide Blocker of the Kv1.3 Channel: Prediction from Free-Energy Simulations and Experimental Confirmation**

**M. Harunur Rashid^1^, Germano Heinzelmann^1^, Redwan Huq^2,3^, Rajeev B. Tajhya ^2,3^, Shih Chieh Chang^4^, Sandeep Chhabra^4^, Michael W. Pennington^5^, Christine Beeton^2^, Raymond S. Norton^4^, Serdar Kuyucak^1^**

^1^ School of Physics, University of Sydney, Sydney, New South Wales, Australia

^2^ Department of Molecular Physiology and Biophysics, Baylor College of Medicine, Houston, Texas, USA

^3^ Graduate Program in Molecular Physiology and Biophysics, Baylor College of Medicine, Houston, Texas, USA

^4^ Medicinal Chemistry, Monash Institute of Pharmaceutical Sciences, Monash University, Parkville, Victoria, Australia

^5^ Peptides International, Louisville, Kentucky, USA

**
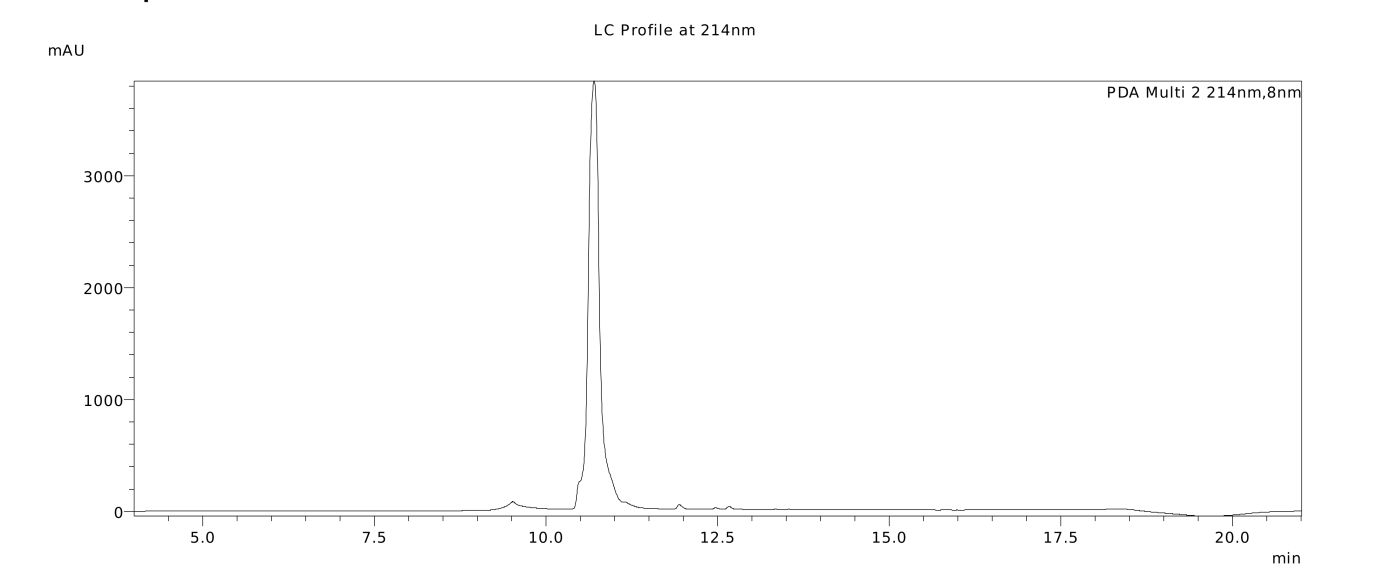
**

**Figure S1. The LC profile of** ShK[K18A].

**
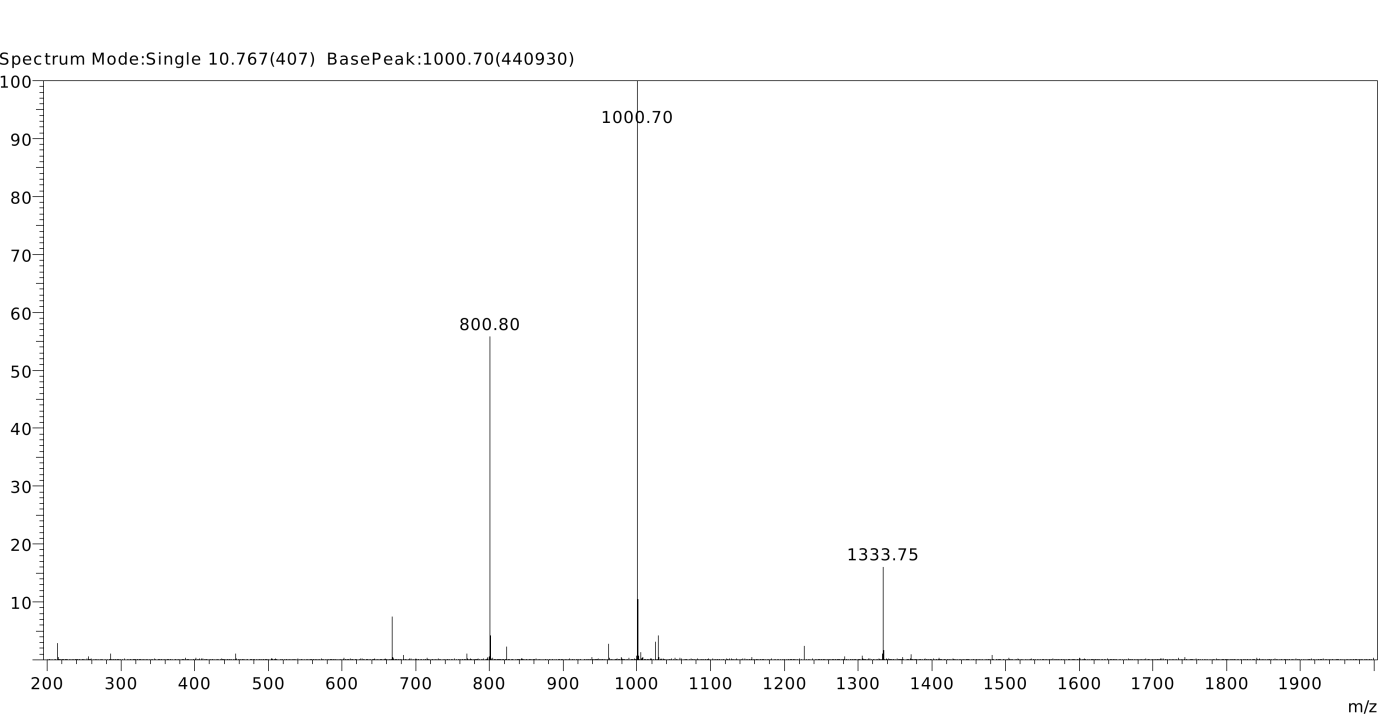
**

**Figure S2. ESI-MS analysis of** ShK[K18A]. The mass of the peptide calculated from the predicted amino acid sequence (3998 Da), compared well to the electrospray mass spectrometry (ESI-MS, 3998 Da).


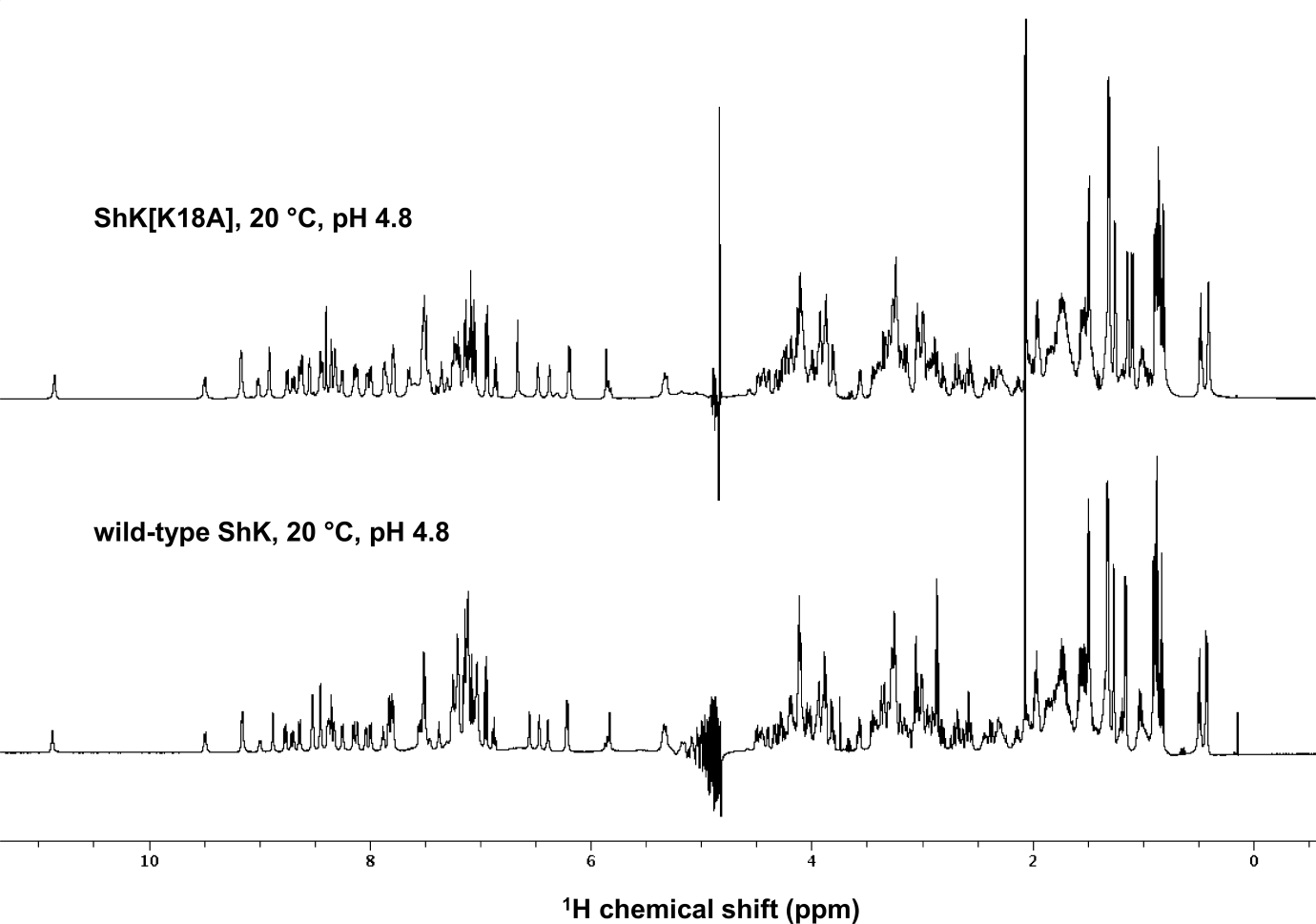


**Figure S3. The 1D ^1^H** NMR spectrum of wild-type ShK and ShK[K18A] at pH 4.8, acquired on a Bruker Avance 600 MHz spectrometer at 20 °C.


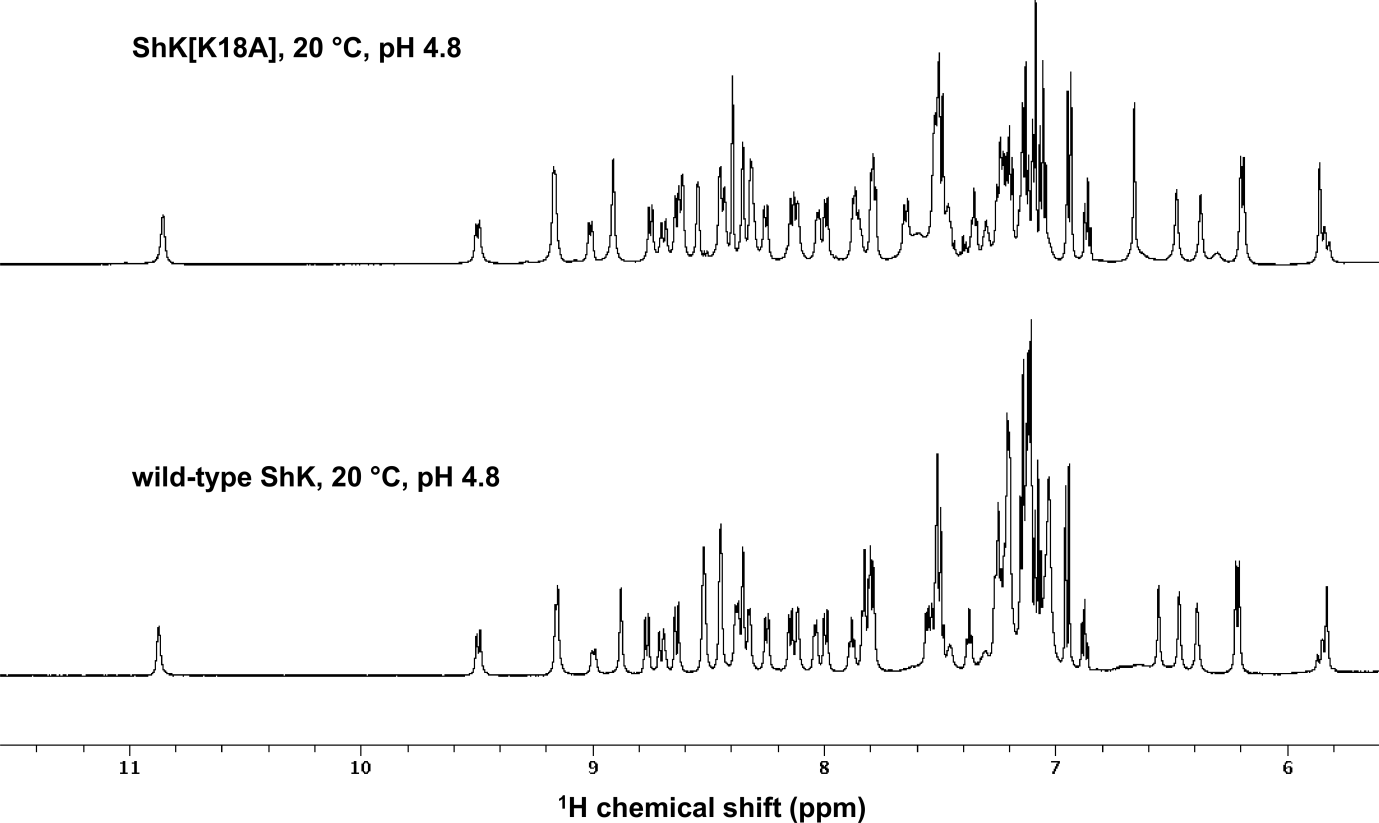


**Figure S4. The** amide and aromatic region **of ^1^H** NMR spectra **of** wild-type ShK and ShK[K18A] at pH 4.8, acquired on a Bruker Avance 600 MHz spectrometer at 20 °C.


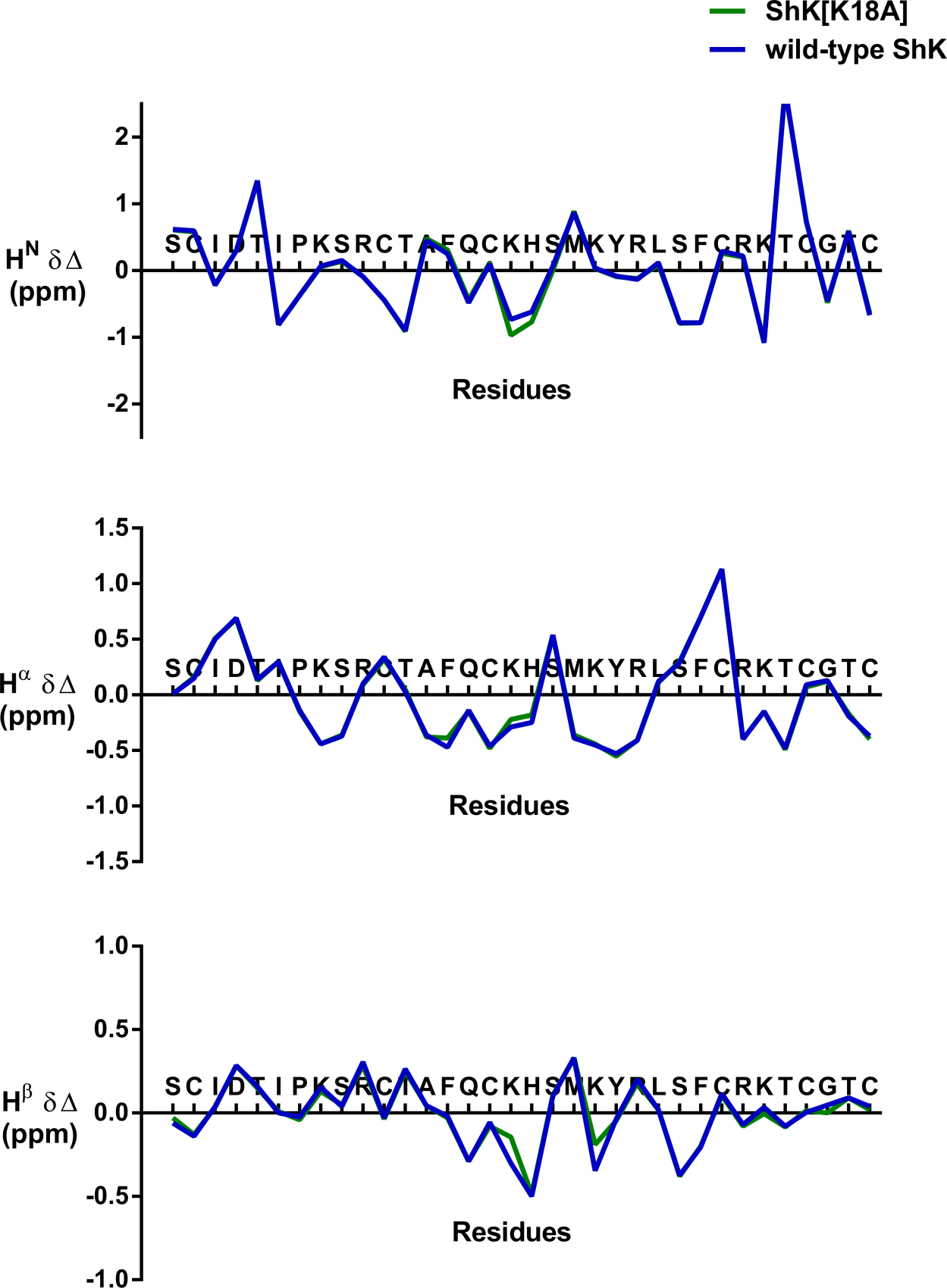


**A**

**B**

**C**

**Figure S5. Deviation from random coil chemical shifts** **(**[**4**](#_ENREF_4)**)** **of the H^N^ (A), H^α^ (B), and H^β^ (C) resonances of** wild-type ShK and ShK[K18A] at 20°C and pH 4.8.


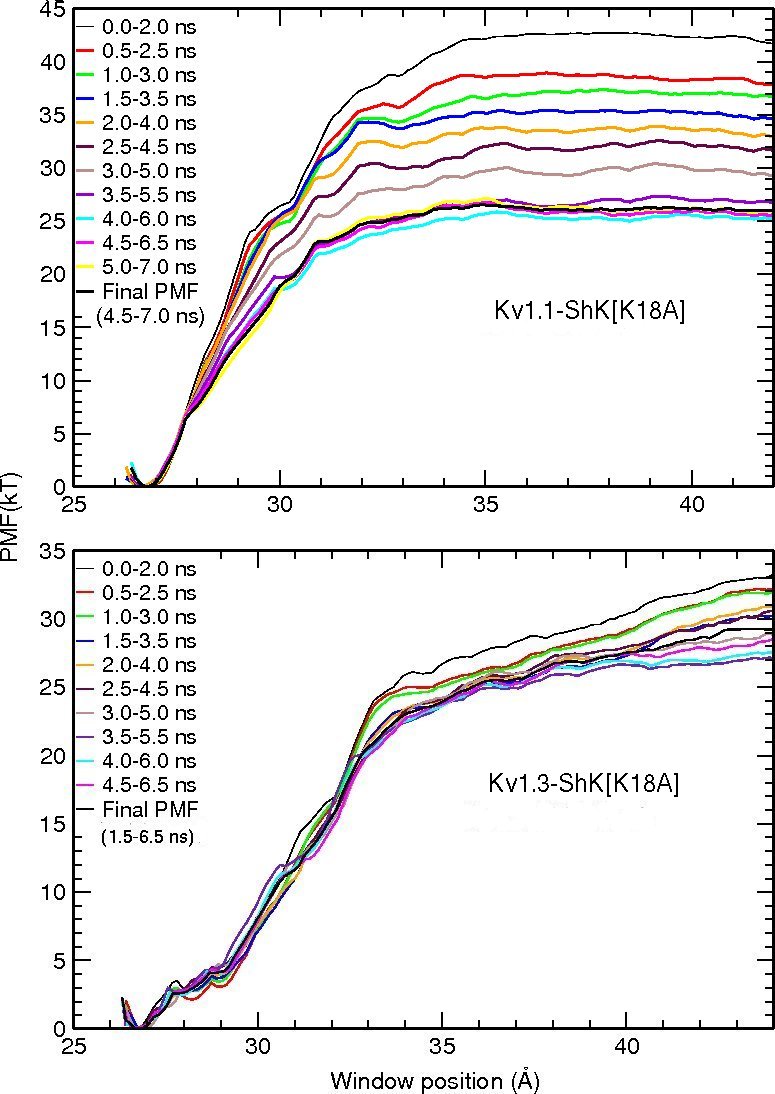


**Figure S6.** **Convergence of the Kv1.x–ShK[K18A] PMFs from 2 ns block data analysis.** To reduce fluctuations, we use a large sampling size (2 ns), which is slid in 0.5 ns steps over the range of the data. In Kv1.1, the PMFs drop monotonically until 3.5-5.5 ns, after which they fluctuate around a base line. In Kv1.3, equilibration occurs earlier, presumably due to the stronger binding. To decide more precisely which part of the data should be discarded for equilibration, we have constructed PMFs from 0.5 ns blocks in Figure S7.


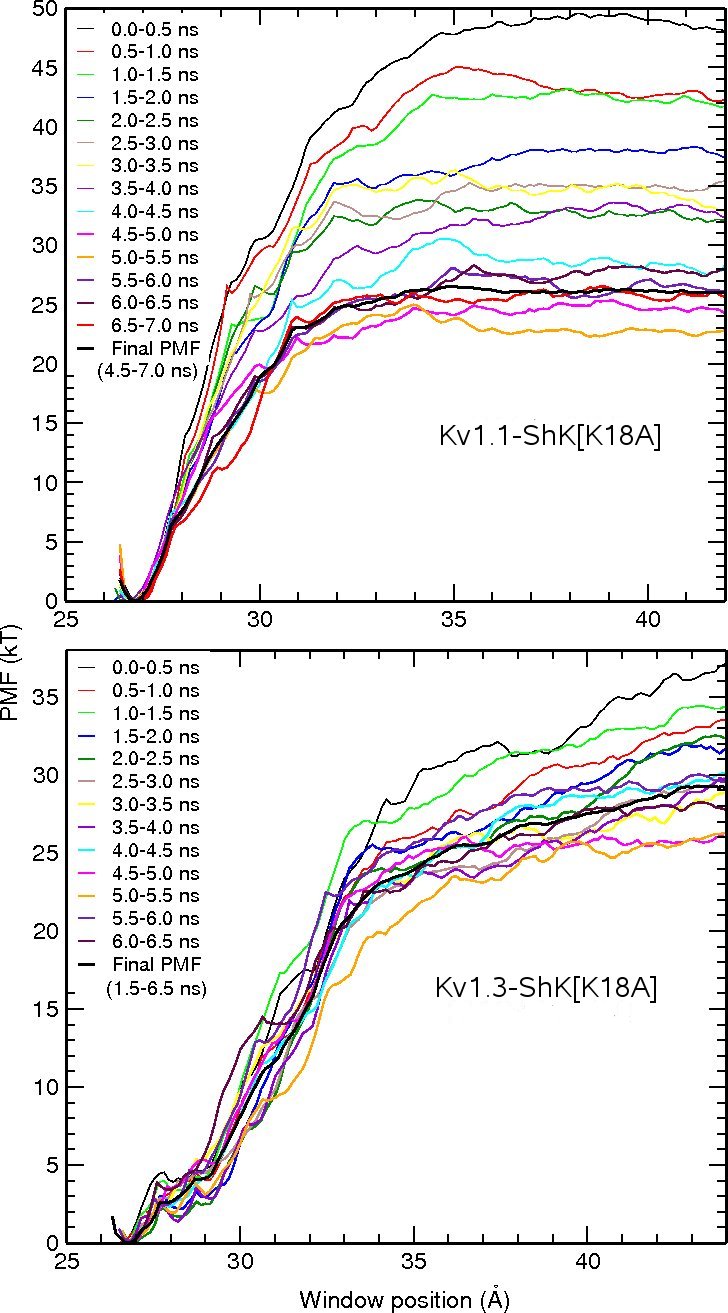


**Figure S7.** **Convergence of the Kv1.x–ShK[K18A] PMFs from 0.5 ns block data analysis.** In Kv1.1, the first 4.5 ns of data are discarded as equilibration, and the final PMF is determined from the last 2.5 ns of data. In Kv1.3, the first 1.5 ns of data are discarded, and the final PMF is determined from the last 5 ns of data.


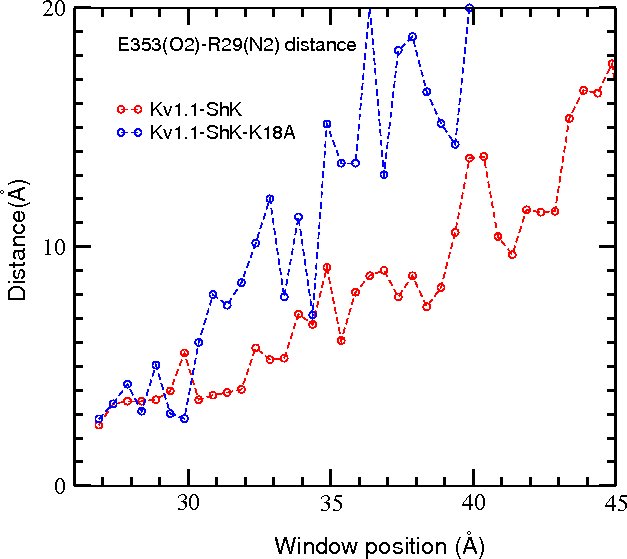


**Figure S8.** **The E353(O_2_)–R29(N_2_) pair distances in ShK and ShK[K18A] PMFs** as a function of the channel-toxin distance in Kv1.1. In the case of ShK[K18A], the pair decouple for *z*>35 Å, which explains the earlier flattening of the corresponding PMF in Figure 4. The bump in the Kv1.1-ShK PMF around *z*=43 Å (Figure 4) can be traced back to the temporary re-engagement of R29 with E353 facilitated by rotation of the toxin.

**Lambda values used in charge/discharge FEP calculations.** 67 exponentially spaced lambda values were used

0.000000000 1.0000004e-03 5.9452732e-03 1.1209489e-02 1.6813217e-02 2.2778355e-02 2.9128211e-02 3.5887597e-02 4.3082927e-02 5.0742315e-02 5.8895691e-02 6.7574915e-02 7.6813901e-02 8.6648751e-02 9.7117893e-02 1.0826224e-01 1.2012533e-01 1.3275353e-01 1.4619617e-01 1.6050579e-01 1.7573831e-01 1.9195323e-01 2.0921393e-01 2.2758785e-01 2.4714678e-01 2.6796716e-01 2.9013033e-01 3.1372291e-01 3.3883708e-01 3.6557097e-01 3.9402905e-01 4.2432252e-01 4.5656975e-01 0.5000000000 5.4343025e-01 5.7567748e-01 6.0597095e-01 6.3442903e-01 6.6116292e-01 6.8627709e-01 7.0986967e-01 7.3203284e-01 7.5285322e-01 7.7241215e-01 7.9078607e-01 8.0804677e-01 8.2426169e-01 8.3949421e-01 8.5380383e-01 8.6724647e-01 8.7987467e-01 8.9173776e-01 9.0288211e-01 9.1335125e-01 9.2318610e-01 9.3242508e-01 9.4110431e-01 9.4925769e-01 9.5691707e-01 9.6411240e-01 9.7087179e-01 9.7722165e-01 9.8318678e-01 9.8879051e-01 9.9405473e-01 9.9900000e-01

1.0000000000

**Lambda values used in charge/discharge TI calculations** (seven point Gaussian quadrature).

0.02544 0.12923 0.29707 0.50000 0.70292 0.87076 0.97455
